# Welcome to Fibrogenesis & Tissue Repair

**DOI:** 10.1186/1755-1536-1-1

**Published:** 2008-10-13

**Authors:** Massimo Pinzani

**Affiliations:** 1 Center for Research, High Education and Transfer, Firenze, Dipartimento di Medicina Interna, Florence, Italy

## 

Fibroproliferative diseases affecting different organs and systems constitute the largest burden and challenge of modern medicine. Nearly 45% of all deaths in the developed world are caused by chronic inflammatory and fibrogenic disorders such as cardiovascular disease, pulmonary fibrosis, progressive kidney disease, systemic sclerosis, liver cirrhosis and inflammatory bowel disease. In addition, chronic inflammation and fibrotic tissue remodelling associated with neo-angiogenesis represent key mechanisms leading to the development of cancer, thus accounting for an additional number of deaths. Regardless of the final clinical outcome, all chronic fibrogenic disorders have a major impact on the quality of life of millions of individuals worldwide and represent a major cost for public health. Despite the massive impact of fibroproliferative diseases on human health, there are no approved treatment options that directly target the mechanisms of fibrosis.

Since research performed over the past thirty years has made it increasingly clear that fibroproliferative disorders often share common mechanisms [1,2], encouraging communication between scientists working in different subspecialties of experimental or clinical medicine and the pharmaceutical industry will help identify common targets and foster the development of new therapeutic strategies. Accordingly, the main aim of *Fibrogenesis & Tissue Repair *is to fill the gap in communication which exists between scientists working on fibrogenesis and tissue repair in different disciplines, allowing them to exchange ideas and to work on common targets.

*Fibrogenesis & Tissue Repair *publishes original and high-quality research articles providing novel insights into the mechanisms, diagnosis and treatment of human diseases characterized by chronic wound healing or fibrogenesis and leading to end-stage organ failure. In particular, the journal focuses on the discovery and characterization of the mechanisms of fibrogenesis and tissue repair common to, or specific for, different organ systems. Along these lines, *Fibrogenesis & Tissue Repair *is divided into five major fibroproliferative disease areas: gastrointestinal and liver, renal, rheumatology and connective tissue, lung, heart and vessels. Particular emphasis is given to those submissions leading to a significant advancement in the fields of genetic predisposition, diagnostic biomarkers and drug discovery. The journal also publishes important advancements in the areas of regenerative medicine, cell therapy and stromal involvement in the genesis of cancer and metastasis. We also solicit reviews in emerging areas of research based on suggestions from members of our Editorial Board [3], whose expertise spans a wide range of disciplines.

Online, open-access publication [4] means that all *Fibrogenesis & Tissue Repair *articles will be available to readers without charge and that movies and animations are only a click away. The benefits of open access are particularly pronounced in a journal such as *Fibrogenesis & Tissue Repair *that aims to promote the rapid exchange of ideas between different specialities.

Manuscripts submitted to *Fibrogenesis & Tissue Repair *will be reviewed by internationally recognized experts in the fields of fibrogenesis, inflammation, and tissue regeneration, selected in part from our Editorial Board. Reviews will be rapid and the suitability of a manuscript for publication will be assessed solely on criteria of scientific excellence. Upon acceptance, articles will be made immediately available online and deposited in PubMed and PubMed Central. Only once a manuscript is accepted for publication will the authors be required to pay an Article Processing Charge to cover the costs of handling their manuscript [5]. The authors are not charged extra for colour figures and there is no page limit.

We sincerely hope that the scientific community involved in the study of fibrogenic diseases and, more broadly, in promoting regenerative medicine will contribute and respond with renewed enthusiasm to this call. The Editors are committed to making this new venture a success, and we hope to receive your contributions in the near future.

## Competing interests

The author declares that they have no competing interests.
